# Arteriovenous malformation in the sigmoid colon of a patient with Cowden disease treated with laparoscopy: a case report

**DOI:** 10.1186/s12893-018-0355-x

**Published:** 2018-04-10

**Authors:** Koichi Inukai, Nobuhiro Takashima, Shiro Fujihata, Hirotaka Miyai, Minoru Yamamoto, Kenji Kobayashi, Moritsugu Tanaka, Tetsushi Hayakawa

**Affiliations:** 1 Department of Surgery, Kariya Toyota General Hospital, 5-15 Sumiyoshi-cho, Kariya, Aichi 448-8505 Japan; 2 Department of laparoscopic hernia center, Kariya Toyota General Hospital, 5-15 Sumiyoshi-cho, Kariya, Aichi 448-8505 Japan

**Keywords:** Arteriovenous malformation, Cowden disease, Laparoscopic surgery

## Abstract

**Background:**

Cowden disease is a genetic disorder associated with a mutation of the *PTEN* gene and is known to be easily complicated by generalized vascular malformations and malignant tumors. However, only a few reports have investigated the relationship between Cowden disease and vascular malformations. We present a case of Cowden disease along with a review of the literature.

**Case presentation:**

The patient was a 48-year-old man who visited our hospital complaining of fresh blood in his stools and shortness of breath. Hematological tests showed the patient had severe anemia. On physical examination, white papules—several millimeters in size—were observed between the patient’s eyebrows. White papules were also observed on the left corner of his mouth and buccal mucosa. An upper gastrointestinal endoscopy showed densely-packed, white, flat protrusions in the esophagus. While lower gastrointestinal endoscopy revealed a mass accompanied by arterial pulsation in the sigmoid colon. A diagnosis of Cowden disease was confirmed and a laparoscopic sigmoidectomy was performed to address the arteriovenous malformations in the sigmoid colon. Post-surgery, the patient had an unremarkable recovery and was discharged 7 days later.

**Conclusions:**

We present a very rare case of Cowden disease with arteriovenous malformations occurring in the colon. Surgical resection is believed to be the first choice for treating congenital arteriovenous malformations of the intestines. However, the arteriovenous malformations in the colon in our patient were treated under laparoscopic guidance, making ours the first report describing laparoscopic treatment of colonic arteriovenous malformations occurring in the inferior mesenteric artery. Thus we demonstrate that laparoscopic treatment of arteriovenous malformations in the intestines is a minimally invasive and can be successfully applied in such cases.

## Background

Cowden disease was first reported in 1963 and is characterized by multiple hamartomas [1]. It is frequently accompanied by a *PTEN* gene mutation, causing increased cell proliferation and vascular neogenesis. As a result, Cowden disease is known to cause mucocutaneous lesions, hamartomas, and generalized vascular malformations, and malignant tumor complications can easily occur.

In this study, we report a patient with a history of Cowden disease who developed a very rare arteriovenous malformation (AVM) of the colon, that was subsequently treated with laparoscopic surgery. Moreover, to date there have only been three reports published in English regarding Cowden disease and vascular malformations. Thus, we also present a review of the literature based on a collection of reports from Japan.

## Case presentation

**Fig. 1:**
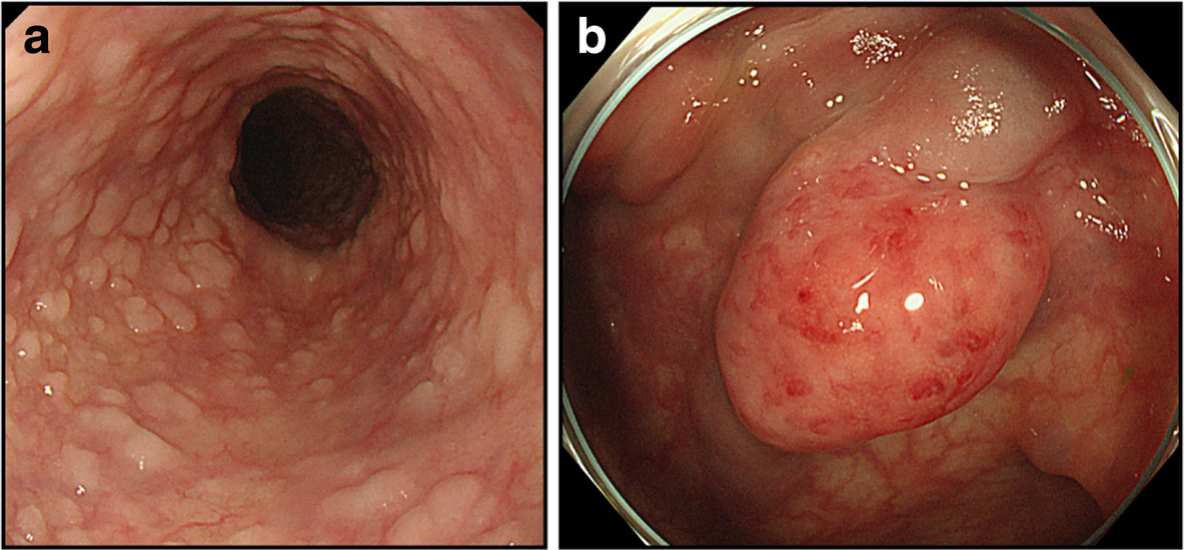
**a** Upper gastrointestinal endoscopy revealed numerous polypoid lesions in the esophagus. **b** Lower gastrointestinal endoscopy showed pulsating abnormal blood vessels exposed on the mucosal surface of the sigmoid colon

**Fig. 2:**
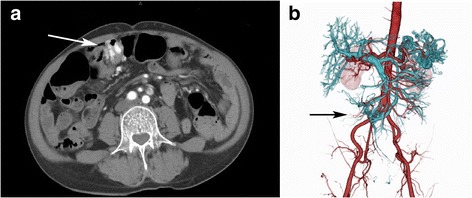
**a** An abdominal contrast computed tomography (CT) scan showed an artery-like vascular malformation in the wall of the sigmoid colon (arrow). **b** CT angiography showed an arteriovenous malformation branching from the inferior mesenteric artery and inferior mesenteric vein (arrow)

A 48-year-old man presented to our hospital complaining of fresh blood in his stools that be had begun experiencing a year earlier, and shortness of breath that had begun half a year earlier. A gastrectomy on the pylorus side was performed for a duodenal ulcer. He reported a family history of cancer, with his mother having been diagnosed with breast cancer. He was hospitalized for a closer examination of anemia as his hemoglobin count was 3.9 g/dL. Physical examination showed anemia in the palpebral conjunctiva. Four white papules were observed between his eyebrows, two papules on the left corner of his mouth, and a papule on the left buccal mucosa. A histological diagnosis of papilloma and fibroma was made based on the papules on the buccal mucosa and between the eyebrows, respectively. A neck ultrasound showed adenomatoid goiter in the thyroid gland. Upper gastrointestinal endoscopy showed multiple flat, white polyps in the esophagus (Fig. 1a), and polyposis was similarly observed in the stomach. Pathophysiological findings revealed that the esophageal polyps were glycogenic acanthosis-like hamartomatous polyps. Lesions in the stomach constituted hyperplastic changes associated with chronic enteritis. Lower gastrointestinal endoscopy showed pulsating abnormal blood vessels exposed on the mucosal surface of the sigmoid colon (Fig. 1b). An abdominal contrast computed tomography (CT) scan showed artery-like vascular malformation in the wall of the sigmoid colon (Fig. 2a). CT angiography showed AVMs branching from the inferior mesenteric artery and inferior mesenteric vein (Fig. 2b).

**Fig. 3:**
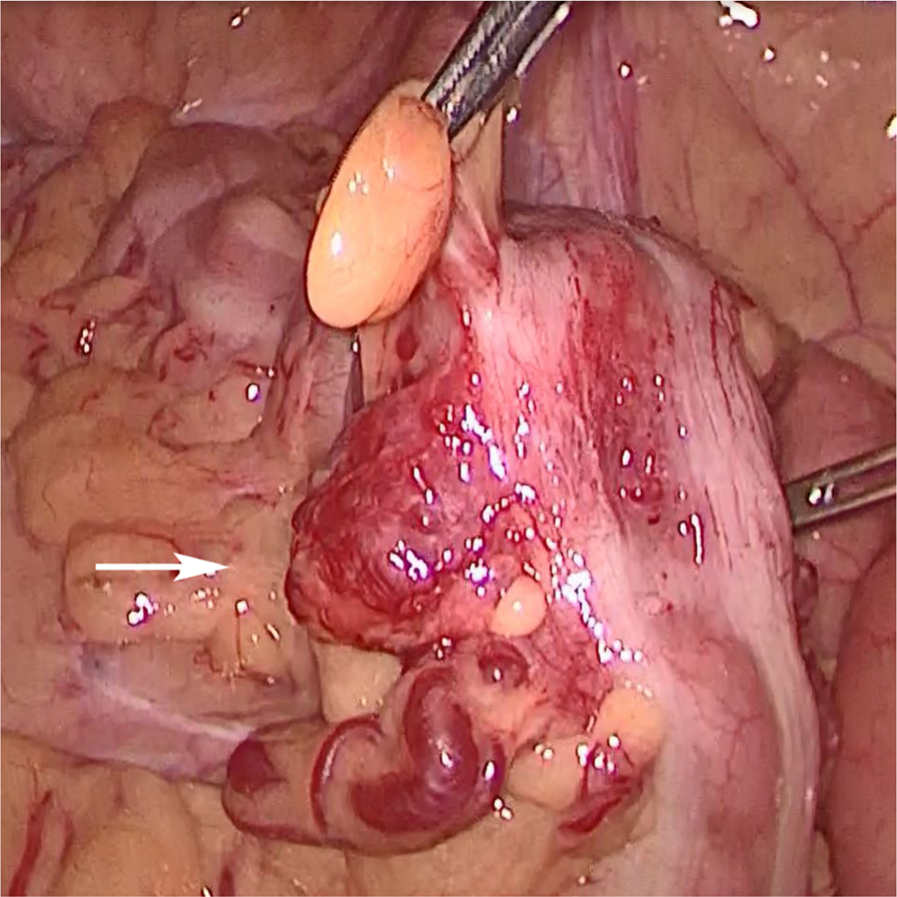
Laparoscopic exploration revealed that the arteriovenous malformation was comprised of dilated mesenteric vessels in the mesocolon region of the sigmoid colon (arrow)

**Fig. 4:**
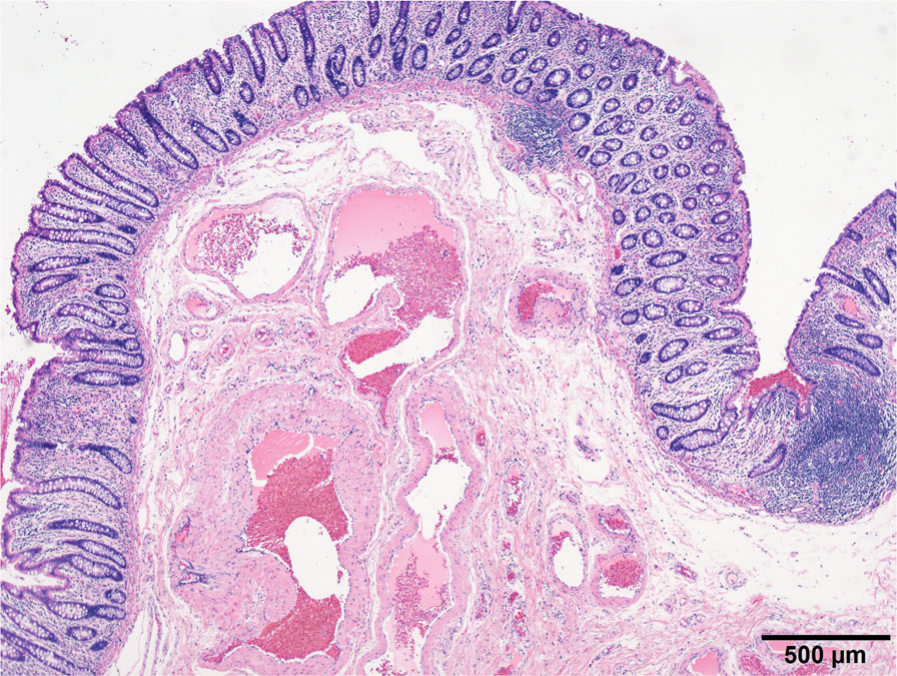
Pathological findings showed vascular malformations that expanded from the submucosal layer to the mesocolon (Hematoxylin and eosin stain)

A clinical diagnosis of Cowden disease was confirmed. Genetic testing was not performed in accordance with the patient’s wishes. The melena was confirmed to be due to the AVM, and a sigmoidectomy was performed under laparoscopic guidance. Laparoscopic observations revealed tortuous blood vessels which expanded on the mesentery of the sigmoid colon (Fig. 3). Intraoperative colonoscopy was used to confirm the position of the lesions from the lumen; the sigmoid colon was mobilized, and the rectum was dissected. Thereafter, the intestines were lifted up from a 5-cm small abdominal incision, the portion with lesions was dissected and an automatic anastomotic device was used to perform anastomosis inside the abdomen. Postoperative pathological findings showed vascular malformations that expanded from the submucosal layer to the mesocolon (Fig. 4). Postoperatively, there were no complications or occurrence of melena, and the patient was discharged 7 days later. Recurrence was not observed 1 year post-surgery.

## Discussion and conclusions

Cowden disease was first reported by Lloyd and Dennis in 1963 and is characterized by multiple facial papules, oral mucosa papilloma, multiple polyposis, and neoplastic lesions in multiple organs [1]. The transmission of Cowden disease is autosomal dominant, and the disease is due to a mutation in the oncosuppressor gene *PTEN* [2]. The diagnostic criteria for Cowden disease was defined by the Genetics/High-Risk Cancer Surveillance Panel of the National Comprehensive Cancer Network and our patient was also diagnosed based on these criteria [3]. Cowden disease, is characterized by polyposis across the entire digestive tract when occurring in the intestines, and its histological types include hamartomatous polyps, hyperplastic changes, gangliocytoma, adenoma [4].

As the risk of gastrointestinal cancer is not elevated with Cowden disease, it is considered acceptable to perform surveillance in the same manner as that performed for a healthy individual. However, generalized cancer surveillance is required and periodic testing for breast cancer and thyroid cancer, in particular, are also required [5].

A few reports have described Cowden disease and vascular lesions [6–8]. Tan et al. studied 26 patients with *PTEN* gene mutations and reported vascular anomalies in 14 patients (56%) [8]. The *PTEN* gene is also expressed in vascular smooth muscle cells, so abnormalities in this gene are considered to cause increased cellular proliferation and angiogenesis, leading to vascular malformations [9].

A search of the literature for Japanese reports on patients with Cowden disease between 1983 and 2017 revealed 122 patients. Forty-two (34.4%) of those patients exhibited vascular malformations (Table 1). Vascular malformations were found in multiple sites in seven of the reported cases. We selected the most clinically significant sites at which vascular malformations occurred in those cases. The results showed that vascular malformations were most common in the extremities. Thus, vascular malformations associated with Cowden disease can appear anywhere, from within the brain to the lower limbs. There were also various feeder blood vessels, from thick blood vessels, such as the common iliac artery, to subcutaneous capillaries.

**Table 1:** Location of vascular malformations in 42 case reports of Cowden disease in the literature

Location	Intracerebral	Neck	Skin on body trunk	Liver	Kidneys	Intestines	Intrapelvic	Extremities	Spine	Total
No. of Cases	1	2	7	10	2	1	4	14	1	42

Most cases were asymptomatic; however, 8 of the 42 listed patients underwent surgery, 5 underwent embolectomy, and 1 underwent radiation therapy. AVM occurring in the digestive tract occasionally causes melena that requires treatment. Generally, gastrointestinal AVM occurs infrequently in the inferior mesenteric artery region [10]. According to our search of the PubMed database, intestinal AVM associated with Cowden syndrome occurred in the small intestine in 1 case [11]. Ours is the first case of AVM reported in the colon, making our report pertinent to the current literature. Moreover, small vascular malformations are easy to overlook and do not induce secondary portal hypertension [12]. Therefore, the actual complication rate might also be underestimated. Vascular malformation occasionally requires treatment in patients with Cowden disease, as in our patient. As such, a full-body evaluation that includes the digestive tract seems important.

Generally, surgical resection, endoscopic treatment, intravascular treatment, etc. are used to treat intestinal AVM but there are no clear guidelines on treatment options. In the present case, AVM was present over a relatively large period and this type of congenital hamartomatous AVM corresponds to Moore classification type 2 and could have infiltrated the full thickness of the intestinal wall [13]. Accordingly, because complete resection of the AVM with endoscopic treatment is not feasible and because we believed that intravascular treatment might cause intestinal ischemia, surgical resection was selected. A few cases of AVM treatment were performed under laparoscopic guidance. A search of PubMed revealed only AVM in the stomach and small intestine; thus, to our knowledge, ours is the first report of the laparoscopic treatment of intestinal AVM occurring in the inferior mesenteric artery region [14–20]. Laparoscopic treatment of AVMs in the intestines can be used for careful observations inside the abdomen if there is no sustained bleeding. Moreover, this modality is minimally-invasive and is, therefore, well indicated for this AVM treatment.

We reported a case of AVM in the sigmoid colon that occurred in a patient with Cowden disease. Patients with Cowden disease may need to undergo full-body examinations for vascular malformation and for deciding on appropriate treatments. Laparoscopy may be considered an effective treatment option for congenital intestinal AVM.

## Abbreviations

AVM: Arteriovenous malformation
CT: Computed tomography
